# Virological success after 12 and 24 months of antiretroviral therapy in sub-Saharan Africa: Comparing results of trials, cohorts and cross-sectional studies using a systematic review and meta-analysis

**DOI:** 10.1371/journal.pone.0174767

**Published:** 2017-04-20

**Authors:** Fabien Taieb, Yoann Madec, Amandine Cournil, Eric Delaporte

**Affiliations:** 1 Emerging Diseases Epidemiology Unit-Institut Pasteur, Paris, France; 2 IRD UMI 233 INSERM U1175 Université de Montpellier, Unité TransVIHMI, Montpellier, France; 3 Direction de la Recherche Clinique et du Développement-Assistance Publique des Hôpitaux de Paris-Hôpital Saint-Louis, Paris, France; University of Toronto, CANADA

## Abstract

**Background:**

UNAIDS recently defined the 90-90-90 target as a way to end the HIV epidemic. However, the proportion of virological success following antiretroviral therapy (ART) may not be as high as the anticipated 90%, and may in fact be highly heterogeneous. We aimed to describe the proportion of virological success in sub-Saharan Africa and to identify factors associated with the proportion of virological success.

**Methods:**

We performed a systematic review and meta-analysis focusing on the proportion of patients in sub-Saharan Africa who demonstrate virological success at 12 and 24 months since ART initiation, as well as at 6 and 36 months, where possible. Programme factors associated with the proportion of virological success were identified using meta-regression. Analyses were conducted using both on-treatment (OT) and intention-to-treat (ITT) approaches.

**Results:**

Eighty-five articles were included in the meta-analysis, corresponding to 125 independent study populations. Using an on-treatment approach, the proportions (95% confidence interval (CI)) of virological success at 12 (n = 64) and at 24 (n = 32) months since ART initiation were 87.7% (81.3–91.0) and 83.7% (79.8–87.6), respectively. Univariate analysis indicated that the proportion of virological success was not different by study design. Multivariate analysis at 24 months showed that the proportion of virological success was significantly larger in studies conducted in public sector sites than in other sites (p = 0.045). Using an ITT approach, the proportions (95% CI) of virological success at 12 (n = 50) and at 24 (n = 20) months were 65.4% (61.8–69.1) and 56.8% (51.3–62.4), respectively. At 12 months, multivariate analysis showed that the proportion of success was significantly lower in cohort studies than in trials (63.0% vs. 71.1%; p = 0.017). At 24 months, univariate analysis demonstrated that the proportion of success was also lower in cohorts.

**Discussion:**

Regardless of the time following ART initiation, and of the threshold, proportions of virological success were highly variable. Evidence from this review suggests that the new international target of 90% of patients controlled is not yet being achieved, and that in order to improve the virological outcome, efforts should be made to improve retention in care.

## Introduction

At the end of 2015, the World Health Organization (WHO) estimated that about 36.7 million people worldwide were living with HIV, with Sub-Saharan Africa the most affected region in the world with 70% of the HIV burden [1].

The 6^th^ Millennium Development Goal called for an unprecedented mobilization to halt and reverse the AIDS epidemic. UNAIDS also set the 90-90-90 target to help end the HIV epidemics (90% of HIV+ diagnosed, 90% of HIV+ treated, 90% of people on treatment achieving supressed viral load). As a result, by the end of 2015, the number of patients receiving antiretroviral therapy (ART) was estimated at more than 15.8 million, an 85% increase since 2010 [2]. This exceeded the WHO goal to provide HIV treatment to 15 million people by the end of 2015 [3]. However, this increase, as well as the recent WHO “treat-all” recommendation [4, 5], challenge the means to evaluate the effectiveness of ART, especially the long-term effectiveness in resource-limited settings.

To enable rapid deployment of ART, many countries have used the WHO public health approach [5], built on the experience of pilot programmes [6], which takes into account the constraints and weaknesses of health systems in low and middle income countries (LMICs): large numbers of patients, limited availability of drugs, and a lack of biological platforms. This approach is characterized by the standardization of 1^st^ and 2^nd^ line ART, the simplification of decision trees and monitoring, the standardization of biological monitoring and the decentralization of care.

In terms of treatment, the preferred choice for adults is a 1^st^ line ART regimen consisting of a backbone of two nucleoside reverse transcriptase inhibitors (NRTI) (tenofovir (TDF) and lamuvidine (3TC) or emtricitabine (FTC)) and one non-nucleoside reverse transcriptase inhibitors (NNRTI) (efavirenz (EFV)) in one daily fixed dose combination. Zidovudine (AZT) may be an alternative to TDF and nevirapine (NVP) may be an alternative to EFV. Since 2010, stavudine (d4T) was replaced by TDF due to its toxicity [7].

The current WHO guidelines for HIV care [4] recommend viral load monitoring at 6 months since ART initiation, at 12 months and then every 12 months. The switch to 2^nd^ line ART is recommended if the confirmed viral load exceeds the threshold of 1000 copies/mL. Although still not widely available in routine care, viral load monitoring is preferred to CD4 count monitoring for the follow-up of HIV patients.

There are numerous studies presenting virological outcomes in patients on ART in sub-Saharan Africa. However the definition of the outcome varies across studies, making the results of individual studies difficult to understand.

Previous systematic reviews of virological outcomes for patients on ART have focused on sub-Saharan Africa and on levels of acquired resistance to antiretroviral drugs [8–10]. In LMICs, summary estimates of viral suppression at different thresholds, including an intention to treat analysis are lacking. Although reviews have been published on virological outcomes, to our knowledge, no meta-analysis on recent data is currently available. This is important given the recent increase in the number of patients now receiving ART.

It is important to evaluate individual sites and programs in terms of virological outcomes. However, the pooling of data from several studies in a meta-analysis can be used to inform countries in the development of wider policy and public health actions to address the burden of HIV/AIDS and to enable WHO to generate international recommendations.

Current WHO guidelines recommend cross-sectional studies to monitor virological efficacy and resistance to ART [11], or the use of cohort studies to assess one of eight early warning indicators of HIV drug resistance: viral load suppression 12 months after ART initiation [12]. However, these two approaches may lead to very different outcomes.

This review and meta-analysis aim to provide updated data on proportions of virological success in adults on ART in sub-Saharan Africa. The objectives were to estimate the proportions of virological success at time points following the beginning of ART, to compare proportions of virological success between different study designs (clinical trials, cohort studies and cross-sectional studies) and to identify factors explaining the heterogeneity between the reported proportions of virological success.

## Methods

We performed a systematic review and meta-analysis in accordance with the Centre for Reviews and Dissemination guidelines [13] and standards of reporting for systematic reviews (PRISMA) [14] (S1 Checklist).

### Search strategy

PubMed, EmBase, Scopus, Web Of Science and Cochrane library were searched for all clinical trials, longitudinal cohort studies and cross-sectional studies on proportions of virological success in adults on ART in sub-Saharan Africa, published in any language between January 1, 2009 and September 30, 2014. We restricted the search to the last 5 years to provide estimates that reflect contemporary care. The search instruction was: (((((HIV OR AIDS[Title/Abstract]))) OR HIV/AIDS[Title/Abstract]) OR ((hiv OR AIDS OR HIV/AIDS[MeSH Terms])))) AND (Africa[Title/Abstract] OR Africa[MeSH Terms]) AND ((((antiretroviral OR ART OR HAART[MeSH Terms]))) OR ((antiretroviral OR ART OR HAART[Title/Abstract])))) AND (((virological OR virologic[MeSH Terms])) OR (virological OR virologic[Title/Abstract]))) AND (("2009/01/01"[PDat]: "2014/05/24"[PDat])). We also manually searched the references of relevant articles to identify studies that might have been missed.

The outcomes of interest were the proportions of patients demonstrating virological success at 6, 12, 24 and 36 months since ART initiation, using on-treatment (OT) and/or intention-to-treat (ITT) analysis.

### Study selection

We considered studies conducted in sub-Saharan Africa that reported virological outcomes for at least one of the time points of interest, strictly in HIV-1 infected treatment-naïve adults (with the definition of the start of adulthood varying between studies: from 14 to 20 years) on 1^st^ line ART, corresponding to the WHO recommendations at that time in Africa.

Studies that did not report virological outcomes at a specific time point or that only included HIV-2 infected populations, paediatric populations, patients on 2^nd^ line ART, or in which more than 20% of patients received non-conventional ART regimen, were not included. Studies reporting virological outcomes on less than 20 patients were also excluded, as such small samples were considered unlikely to provide accurate information and were likely subject to significant biases.

All articles were independently reviewed by two of the authors (FT and YM). The titles and abstracts of all identified articles were reviewed to determine the eligibility of full text papers for inclusion. The remaining full-length articles were retrieved and read independently by the two authors to determine whether to include them in the meta-analysis. Results were compared and discrepancies in opinion between authors as to whether studies should be included were resolved by discussion.

### Data extraction

To extract relevant data from the selected articles, a standardized collection form was prepared. Data extraction and validity assessment were carried out independently and in duplicate by two of the authors (FT and YM), and any discrepancies resolved by discussion. The authors contacted the corresponding authors of individual studies to retrieve any missing data. The completed forms and resulting electronic database were checked by a third author (AC) for data accuracy and quality.

For clinical trials, each study arm was considered as an independent study and outcomes were reported independently [15–17]. For longitudinal cohort studies and cross-sectional studies, results which were reported by groups were also regarded as independent studies and outcomes were reported independently.

For all studies, we recorded the threshold at which virological success had been defined, the number of patients evaluated, and the number/proportion of patients demonstrating virological success, enabling OT analysis. For ITT analysis, death, loss to follow-up (LTFU), and the switching to second-line ART prior to the virological evaluation were considered as virological failures, while patients who withdrew from the study or who transferred out of the study prior to the virological evaluation were not considered. The ITT result was not included if the proportion of patients not considered for unknown reasons exceeded 40%.

The quality of the studies included in this meta-analysis was assessed based on the STROBE (Strengthening the reporting of observational studies in epidemiology) checklist [18]. Availability of information regarding the description of the study design, the setting, eligibility criteria, details on virological evaluation, sample size calculation, number of participants at each stage of the study, the reasons for non-participation at each stage of the study, the presentation of baseline characteristics of study participants, reporting of the number of patients in virological success and discussion of the limitations of the study were assessed for all studies eligible for inclusion in the meta-analysis.

### Statistical analysis

The overall proportion of patients demonstrating virological success was estimated using a random effect model, following the method of DerSimonian and Laird [19], without weight, and using the *metan* command in Stata (Stata Corp, College Station, Texas, USA). Heterogeneity was indicated if the Cochran test was significant at the level of 0.05 and/or if the I-square statistic exceeded 0.50.

The proportion of patients demonstrating virological success was estimated as the number of patients in virological success divided by the number of patients with a viral load measurement (OT analysis), or as the number of patients in virological success divided by the number of patients who should have reached the evaluation time point minus the number of transfers/withdrawal before the evaluation time point (ITT analysis). Substitution to 2^nd^ line ART before the evaluation time-point was regarded as virological failure. The standard deviation for all proportions was obtained using a Gaussian approximation.

Due to the variability in reporting of the virological thresholds, virological success at <50 copies/mL included all results at 20, 40 and 50 copies/mL thresholds. In the same way, reporting of results at 100, 150, 200, 400 and 500 copies/mL thresholds were combined and considered as virological success at 400 copies/mL. Virological failure was defined as >1000 copies/mL, in accordance with the latest WHO recommendations [4]. We also assessed the proportion of virological failure at 5000 copies/mL, which corresponds to the 2010 WHO recommendations [7].

The effect of the following factors on the level of virological success was investigated: beginning of enrolment (i.e. ART initiation) (≤2005, ≥2006), end of enrolment (≤2005, 2006–2009, ≥2010), study design, study region [20], type of site (public vs. other), number of sites, decentralized setting (if the authors mentioned “decentralized”, “tertiary level”, “district level” or “rural” in the method section), general HIV population vs. specific population (i.e. HBV or HCV co-infected patients, TB-infected patients, or Kaposi-diagnosed patients), and tracking of LTFU patients (as reported in the study).

We wanted to evaluate whether previous viral load monitoring affected the level of virological success at the time of evaluation. Hence, we defined a binary indicator of previous routine virological evaluation and we included details of viral load monitoring if performed routinely as part of the study.

The effect of factors describing the population at baseline were also investigated once categorised based on the median across all studies: proportion of women, age, proportion of patients in WHO stage 3 or 4, median CD4 level, median viral load level, first line ART (EFV or NVP-based). The effect of all these factors was investigated using meta-regression and p-values were estimated through a permutation test. To ensure that convergence of the permutation test was achieved, we verified that estimates obtained using 10,000, 30,000 and 50,000 iterations were similar. Factors which presented a p-value <0.20 in univariate analysis were considered in the multivariate model. Using a backward stepwise procedure, we identified factors which remained independently and statistically significantly associated with the outcome. To assess the effect of publication bias, funnel plots representing the proportion of patients in a study in virological success and the sample size of the study were examined [21].

For the ITT analysis, we further distinguished two groups within the general population: studies which enrolled all patients without a minimum follow-up duration and studies which enrolled patients with a minimum follow-up of 6 months. This consideration was important as it has been shown that mortality is particularly high within the first 6 months following ART initiation [22, 23].

## Results

### Selection of studies

**Fig 1:**
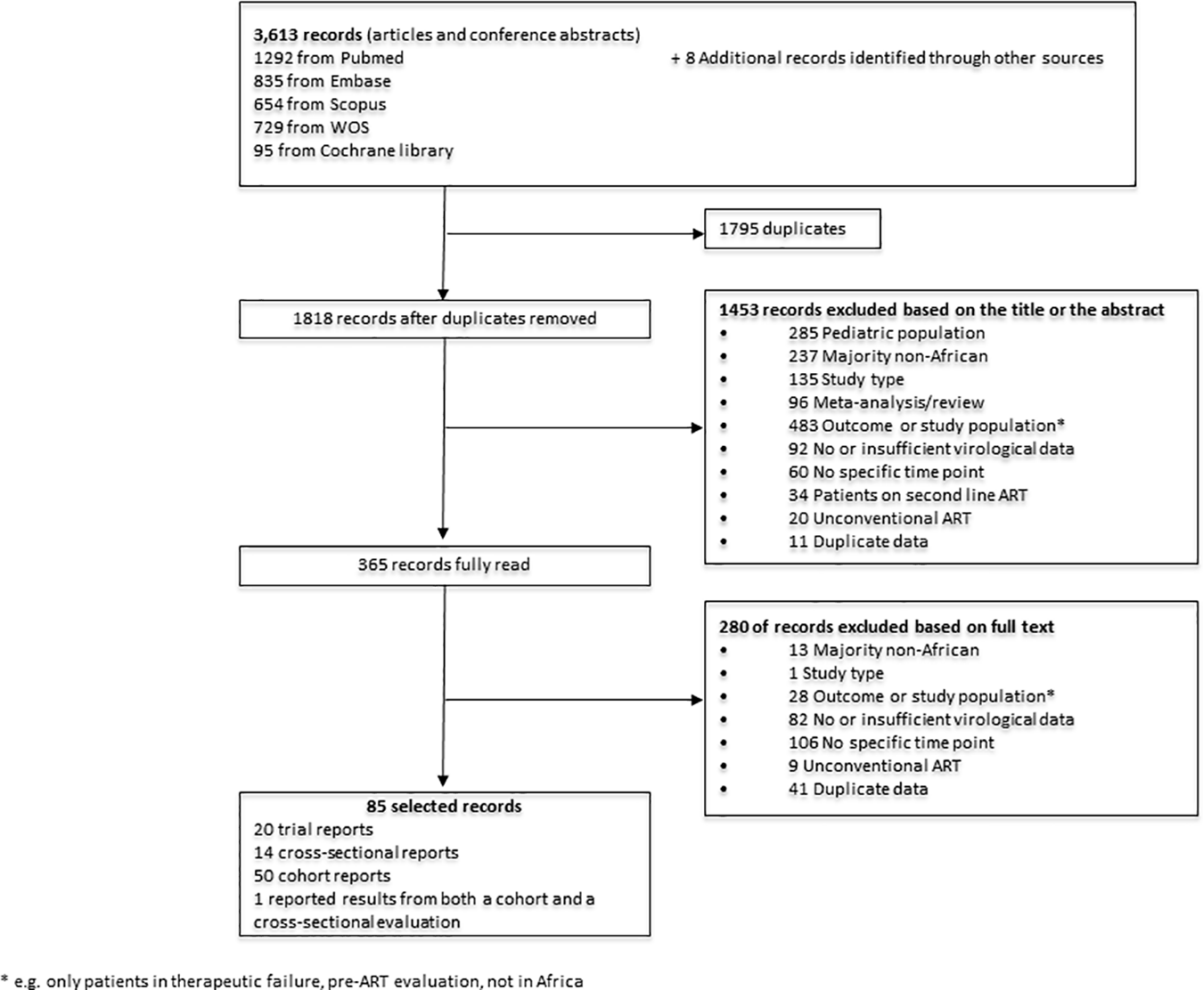
Flow chart of study selection.

We identified 3613 articles reporting on proportions of virological success following ART initiation. After removing duplicate publications and clearly irrelevant publications, we retrieved 365 articles which were reviewed independently for eligibility. 85 articles identified in the original search contributed to the meta-analysis: 20 articles on clinical trials, 50 articles on cohort studies, 14 articles on cross-sectional studies, and one article reported results from both a cohort and a cross-sectional evaluation [24]. Fig 1 indicates the process of study selection.

To evaluate the quality of the 85 articles considered in this meta-analysis, we selected the items from the STROBE statement that we considered relevant (S1 Table).

### Characteristics of studies

Of the 20 articles on clinical trials included in the review and meta-analysis, two articles reported on the same trial, but reported results at different time points following ART initiation [25, 26]. Among these 19 clinical trials, 8 had a single arm [25, 27–33], 10 had two arms [34–43], and another one had two arms but reported only aggregated results from the two arms [44]. This gave 29 independent study populations. Most trials compared care conditions, and the treatment received in the different study arms were therefore comparable, although some trials considered compared ART regimen.

Of the 15 articles on cross-sectional studies, one was conducted across 5 countries at two different time points in each country [45], three were conducted in one country at two different time points [46–48], two were conducted in one country at one time point but among two different groups [49, 50], two were conducted in one country and at three different time points [51, 52], and the remaining seven were conducted at a single time point [24, 53–58]. This gave 33 distinct study populations.

Of the 51 articles on cohort studies, three articles were based on the same cohort but reported results at different time points [59–61], and two other articles reported on a same cohort [62, 63]. Of the 48 independent cohorts, 31 reported results from the overall cohort [24, 62, 64–92], while 17 reported results from two different groups within the cohort [59, 93–108], although for two of these studies, only one group was considered in our meta-analysis due to sample size restriction [100, 107]. This gave 63 distinct populations.

This meta-analysis is therefore based on 125 distinct populations, which accounted for a total of 156,798 patients, with individual study sizes ranging from 23 to 47,285 patients.

Across the 125 populations, the median proportion of women was 65.7% and individual studies ranged from 0 to 100% proportion of women. The populations were quite homogenous in terms of age, with the median/mean age in individual studies ranging from 29 to 42 years, and a median age across studies of 36 years. In all but one of the studies, ART initiation followed the WHO guidelines [109]. This study only enrolled patients with CD4 count >350 cells/mm^3^ [30]. The median proportion of patients across all studies classified as stage 3–4 according to WHO guidelines was 66.3%. The mean/median CD4 level at ART initiation in individual studies ranged from 33 to 569 cells/mm^3^ and the median across all studies was 134 cells/mm^3^. Baseline viral load was available for 58 (46.4%) of the study populations. The median viral load level at baseline in individual studies ranged from 4.0 to 6.2 log copies/mL, and across all studies the median was 5.1 log copies/mL. As we only selected studies in which the first line ART fulfilled WHO recommendations, all patients had initiated an ART regimen that contained either 3TC or FTC. For exposure to NNRTI, the median (IQR) proportion of patients across all studies receiving EFV and NVP was 34.5% (1.1–62.5) and 60.0% (31.6–95.0), respectively.

These 125 study populations enabled the evaluation of the proportion of virological success at 6 months (n = 54), 12 months (n = 84), 24 months (n = 41) and 36 months (n = 15) after ART initiation.

Of the 125 study populations, the proportion of virological success was available for using an on-treatment approach in 119 (95.2%). Using an intention-to-treat (ITT) approach, results on the proportion of virological success could be obtained from 76 (60.8%) study populations.

### Virological success at 12 months

The results of 84 evaluations of virological success at 12 months after ART initiation were considered: 22 from clinical trials, 47 from cohort studies, and 15 from cross-sectional studies.

**Fig 2:**
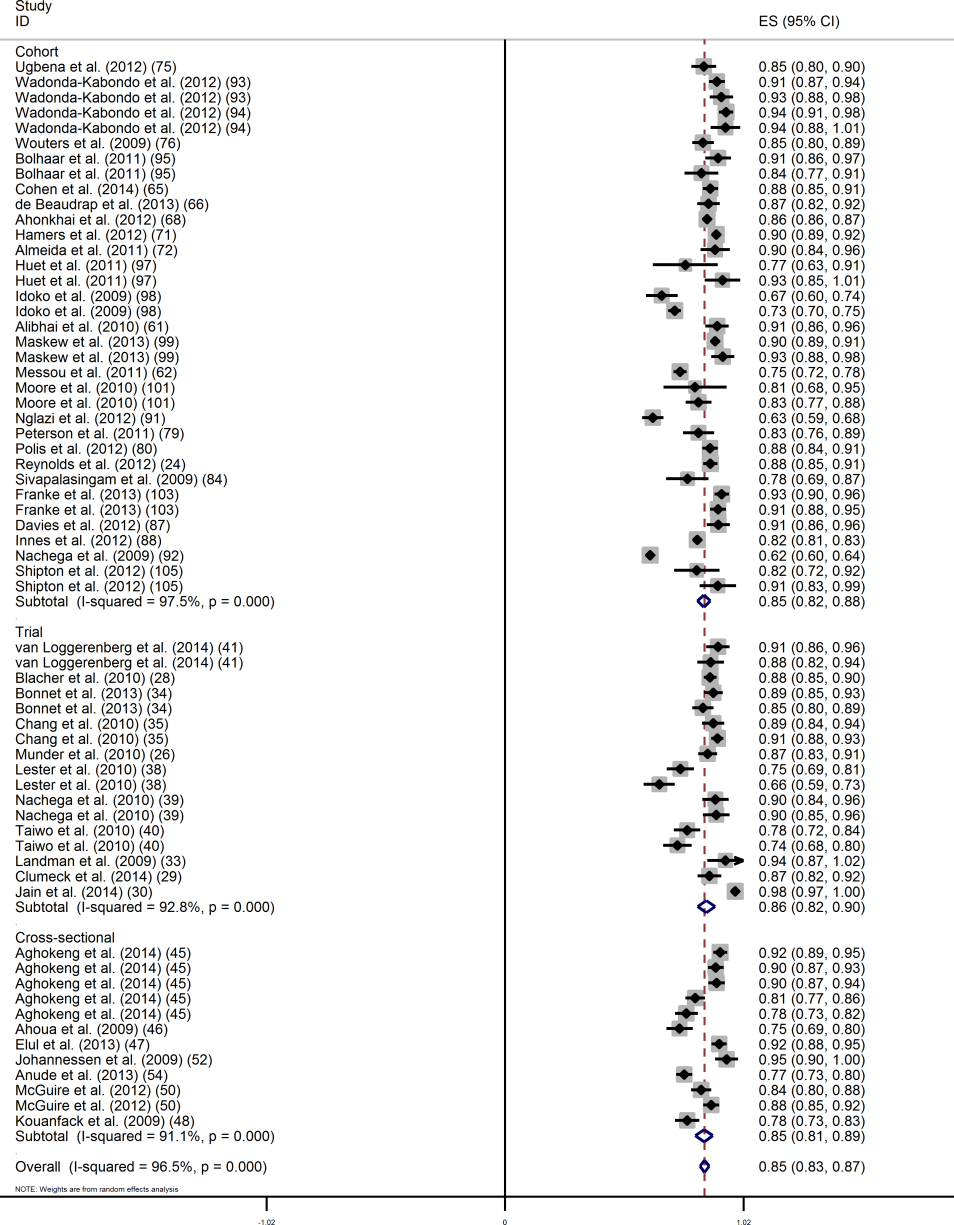
On treatment analysis of the proportion of virological success (400 copies/mL) at 12 months of ART by study design.

Considering OT results only, 64 evaluations of virological success were available at the threshold of 400 copies/mL and the overall proportion (95% confidence interval (CI)) of patients in virological success was 85.3% (83.3–87.3) (Fig 2). The Cochran test (p<0.001) and the I-square statistic (96.6%) indicated large between-study heterogeneity.

In univariate analysis, we did not find evidence that the proportions differed by study design (Table 1). The proportions (95% CI) of patients in virological success in clinical trials, cohort studies and cross-sectional studies were 86.1% (82.3–89.8), 85.1% (82.3–87.8) and 85.1% (81.3–88.9), respectively. The proportion of virological success was significantly higher in studies that were set in the public sector, that were in decentralised settings, and that had a higher proportion of patients on EFV-based ART (Table 1). On the other hand, the proportion of virological success was lower in studies in the general population with >6 months of follow-up, compared to studies without this restriction. When combining all factors in a multivariate model, no factor remained independently associated with the proportion of virological failure.

**Table 1:** Virological success (<400 copies/ml) at 12 months–on-treatment analysis.

				Univariate	Multivariate	
				analysis	analysis	
Variables	Categories	N	Rate (95% CI)	(p)	Full model (p)	Reduced model (p)
Study design	Cohort	35	85.1 [83.2–87.8]	Ref		
	Trial	17	86.1 [82.3–89.8]	0.90		
	Cross sectional	12	85.1 [81.3–88.9]	0.99		
Beginning of enrolment	≤2005	34	84.4 [81.5–87.4]	Ref		
	≥2006	30	86.3 [83.8–88.8]	0.36		
End of enrolment	≤2005	11	85.6 [82.2–88.9]	Ref		
	2006–2009	45	84.5 [82.2–86.9]	0.92		
	≥2010	7	89.6 [84.9–94.3]	0.46		
Region	Western Africa	16	81.5 [77.4–85.5]	0.11		
	Eastern Africa	25	87.3 [84.8–89.8]	Ref		
	Central Africa	3	85.3 [78.2–92.4]	0.99		
	Southern Africa	16	86.6 [84.1–89.1]	0.99		
	Several region	2	76.2 [0.48–1.00]	0.18		
Type of site	Public sector	51	86.4 [84.5–88.3]	Ref	Ref	Ref
	Other	10	80.2 [73.2–87.3]	0.04	0.76	0.04
Number of sites	1	35	85.1 [81.9–88.3]	Ref		
	≥2	20	85.9 [83.4–88.5]	0.76		
Decentralised setting	No	39	83.9 [80.8–86.9]	Ref	Ref	
	Yes	15	89.6 [85.7–93.5]	0.04	0.35	
	Both	7	82.4 [78.4–86.5]	0.79	0.30	
Population selected	General	44	85.9 [84.3–87.5]	Ref	Ref	
	General, with FU >6 months	7	77.5 [68.9–86.1]	0.01	0.43	
	Specific	7	89.0 [86.5–91.5]	0.44	0.61	
VL monitoring before evaluation	No	31	85.5 [82.1–88.8]	Ref		
	Yes	33	85.2 [82.7–87.6]	0.86		
Tracking of LTFU patients	No	49	85.4 [83.0–87.9]	Ref		
	Yes	15	85.0 [81.1–8.8]	0.85		
Median age at ART initiation (years)	≤36	32	84.7 [82.4–87.0]	Ref		
	>36	26	85.8 [81.4–90.2]	0.60		
Proportion of women	≤65	34	86.8 [83.7–89.9]	Ref		
	>65	30	83.7 [81.3–86.1}	0.14		
Proportion of patients in WHO stage 3–4	≤69	25	84.4 [82.0–86.8]	Ref		
	>69	18	87.9 [85.3–90.6]	0.23		
Median CD4 at ART initiation (cells/mm^3^)	≤135	30	85.9 [84.1–87.7]	Ref		
	>135	27	84.4 [79.7–89.1]	0.84		
Median VL at ART initiation (log copies/mL)	≤5.2	14	81.6 [77.3–85.8]	Ref		
	>5.2	22	87.5 [84.8–90.1]	0.09		
Proportion of EFV exposed patients	≤40	22	82.0 [77.2–86.9]	Ref	Ref	
	>40	20	87.4 [84.8–90.0]	0.04	0.99	
Proportion of NVP exposed patients	≤60	23	85.2 [81.4–89.0]	Ref		
	>60	19	83.1 [79.9–86.4]	0.41		

Using an OT approach, 19 evaluations of virological success were available at 40 copies/mL threshold, 22 evaluations of virological success at 1000 copies/mL threshold and 12 evaluations of virological success at 5000 copies/mL threshold. The overall proportions (95% CI) of virological success at these thresholds were 76.3% (71.6–81.0), 87.0% (84.6–89.5) and 90.8% (87.9–93.7), respectively (S1 Fig).

**Fig 3:**
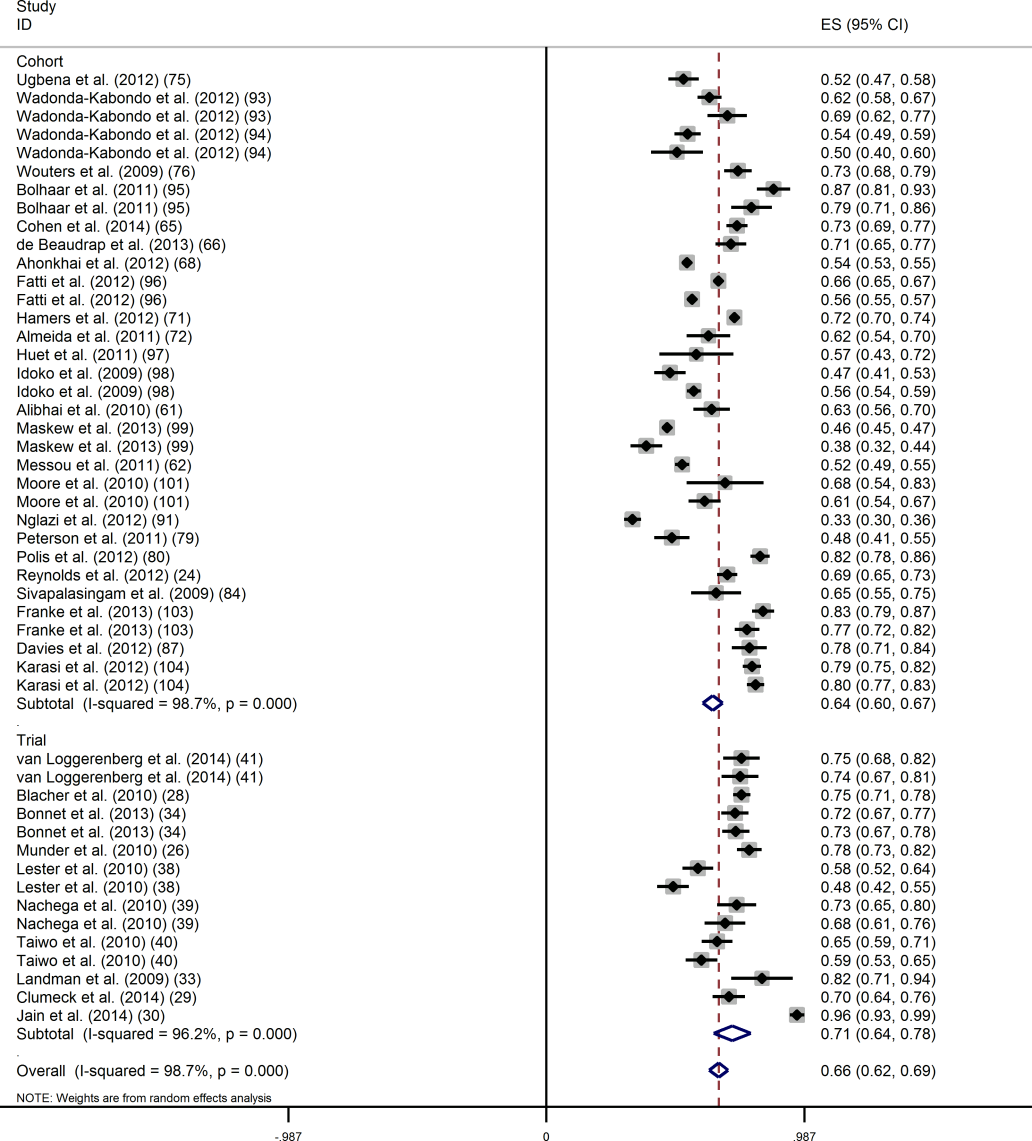
Intention-to-treat analysis of the proportion of virological success (400 copies/mL) at 12 months of ART by study design.

Using an ITT approach, 50 evaluations were considered at the threshold of 400 copies/mL, and the overall proportion of virological success (95% CI) was 65.9% [62.3–69.5] (Fig 3). Again, the Cochran test (p<0.001) and the I-square statistic (98.7%) indicated large between-study heterogeneity. In univariate analysis, the proportion of virological success (95% CI) was significantly lower in cohort studies than in clinical trials (63.6% (59.8–67.5) versus 71.1% (64.0–78.2);p = 0.04) (Table 2). The proportion of virological success was significantly higher in studies that enrolled patients who had initiated ART more recently (≥2006), and in studies with a higher median age at ART initiation. In multivariate analysis, the proportion of virological success remained significantly lower in cohort studies.

**Table 2:** Rate of virological success (<400 copies/ml) at 12 months–intention to treat analysis.

				Univariate	Multivariate	
				analysis	analysis	
Variables	Categories	N	Rate (95% CI)	(p)	Full model (p)	Reduced model (p)
Study design	Cohort	35	63.6 [59.8–67.5]	Ref	Ref	Ref
	Trial	15	71.1 [64.0–78.2]	0.037	0.07	0.039
Beginning of enrolment	≤2005	28	62.1 [58.2–66.1]	Ref	Ref	
	≥2006	22	69.4 [63.9–74.9]	0.031	0.82	
End of enrolment	≤2005	7	70.6 [65.5–75.7]	0.22		
	2006–2009	35	62.1 [57.9–66.2]	Ref		
	≥2010	7	74.4 [64.3–84.5]	0.05		
Region	Western Africa	10	58.4 [53.5–63.2]	0.09		
	Eastern Africa	20	68.6 [62.8–74.3]	Ref		
	Central Africa	1	69.9 [63.6–76.1]	1.00		
	Southern Africa	16	62.8 [57.8–67.8]	0.32		
	Several region	1	72.0 [70.3–73.8]	0.99		
Type of site	Public sector	37	65.5 [59.8–71.2]	Ref		
	Other	8	62.4 [57.1–67.7]	0.57		
Number of sites	1	26	67.1 [59.8–74.3]	Ref		
	≥2	16	63.1 [55.6–70.6]	0.36		
Decentralised setting	No	27	66.4 [60.3–70.4]	Ref		
	Yes	11	68.8 [54.5–83.1]	0.80		
	Both	7	58.4 [52.9–63.9]	0.25		
Population selected	General	33	65.1 [60.7–69.4]	Ref		
	General, with FU >6 months	5	59.8 [51.8–67.8]	0.64		
	Specific	6	66.6 [54.3–78.9]	0.96		
VL monitoring before evaluation	No	18	64.4 [60.4–68.5]	Ref		
	Yes	32	66.0 [60.0–72.1]	0.69		
Tracking of LTFU patients	No	36	66.5 [61.2–71.8]	Ref		
	Yes	14	62.7 [57.1–68.4]	0.40		
Median age at ART initiation (years)	≤36	25	59.9 [55.3–64.4]	Ref	Ref	Ref
	>36	18	68.4 [64.2–72.5]	0.029	0.13	0.10
% women	≤65	21	64.7 [58.3–71.1]	Ref		
	>65	27	64.9 [60.7–59.2]	0.94		
% WHO stage 3–4	≤65	15	62.4 [53.5–71.2]	Ref		
	>65	16	68.8 [63.3–74.3]	0.82		
Median CD4 level	≤130	26	63.0 [59.1–66.9]	Ref		
	>130	19	67.5 [61.3–73.6]	0.21		
Median VL level	≤5.2	12	61.3 [53.9–68.8]	Ref		
	>5.2	18	67.2 [61.5–72.9]	0.22		
% with EFV	≤40	15	64.6 [58.6–70.6]	Ref		
	>40	15	65.1 [58.9–71.4]	0.92		
% with NVP	≤60	16	64.3 [58.3–70.2]	Ref		
	>60	14	65.6 [59.7–71.5]	0.80		

Using an ITT approach, 15 evaluations of virological success were available at 40 copies/mL threshold, 12 evaluations of virological success were available at 1000 copies/mL and 3 evaluations of virological success were available at 5000 copies/mL threshold. The overall proportions (95% CI) of virological success at these thresholds were 54.9% (40.9–69.0), 66.5% (60.8–72.2) and 76.9% (71.2–82.6), respectively (S1 Fig).

### Virological success at 24 months

**Fig 4:**
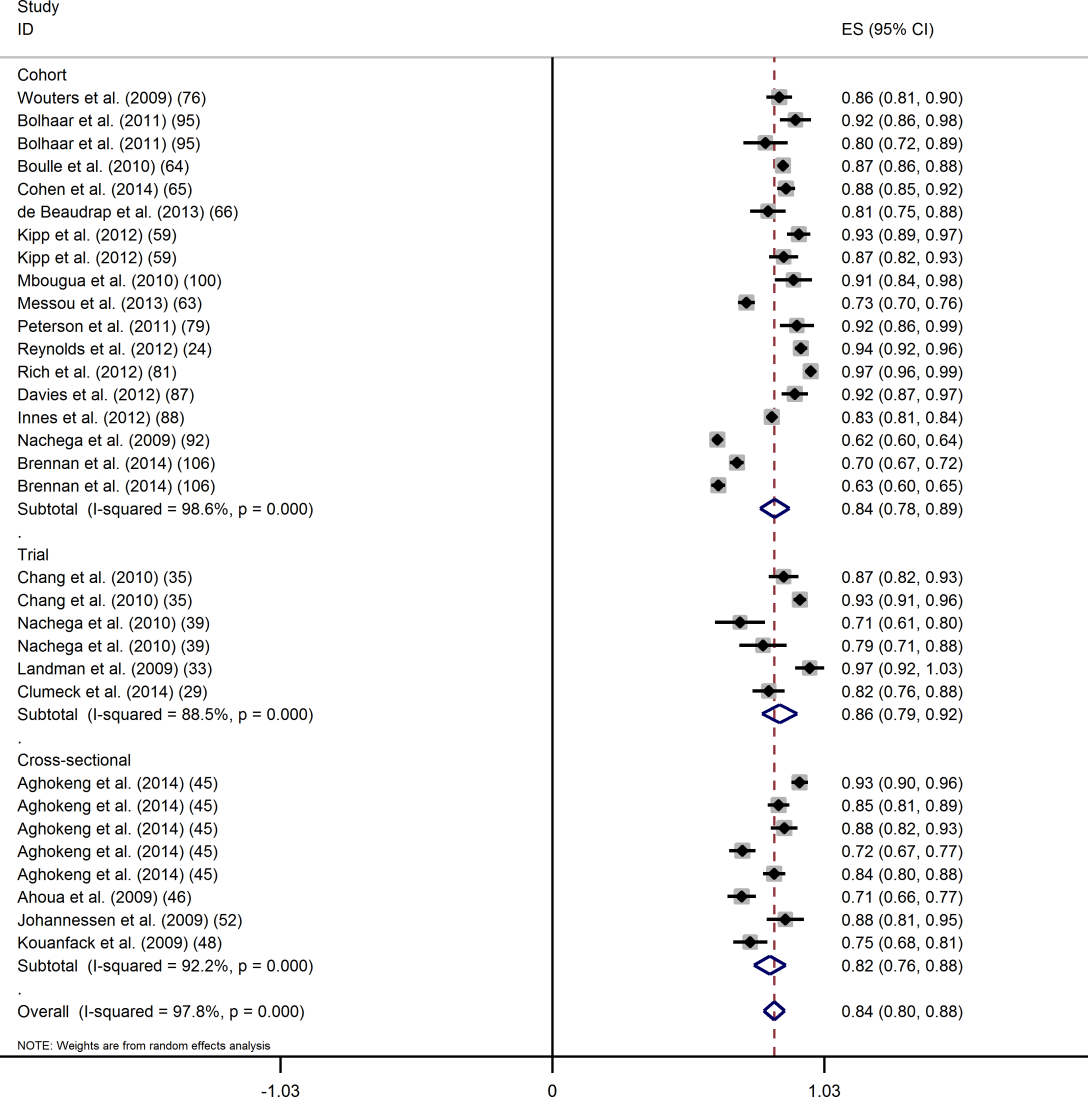
On treatment analysis of the proportion of virological success (400 copies/mL) at 24 months of ART by study design.

Using an OT approach, 32 evaluations of virological success at 24 months after ART initiation at the threshold of 400 copies/mL were available. The overall proportion of virological success (95% CI) was 83.7% (79.8–87.6) (Fig 4). The Cochran test (p<0.001) and the I-square statistic (97.8%) indicated large between-study heterogeneity.

In univariate analysis, we did not find evidence that the proportions differed by study design (Table 3). The proportions of virological success (95% CI) in clinical trials, cohort studies and cross-sectional studies were 85.8% (79.1–92.4), 83.9% (78.3–89.5) and 82.1% (76.1–88.1), respectively. The proportion of virological success was found to be significantly higher in public sector sites compared to other sites, in studies with younger populations, and in studies with a higher proportion of women. The proportion of virological success also tended to be higher in studies with lower viral load at ART initiation. When combining all factors in a multivariate model, the only factor that remained significantly associated with a higher proportion of virological success was when the study was set in the public sector.

**Table 3:** Rate of virological success (<400 copies/ml) at 24 months–on-treatment analysis.

				Univariate	Multivariate	
				analysis	analysis	
Variables	Categories	N	Rate (95% CI)	(p)	Full model (p)	Reduced model (p)
Study design	Cohort	18	83.9 [78.3–89.5]	Ref		
	Trial	6	85.8 [79.1–92.4]	0.88		
	Cross-sectional	8	82.1 [76.1–88.1]	0.94		
Beginning of enrolment initiation	≤2005	21	85.3 [80.6–89.9]	Ref		
	≥2006	11	80.8 [73.9–87.7]	0.21		
End of enrolment	≤2005	6	85.0 [76.3–93.8]	1.00		
	2006–2009	20	85.0 [80.3–89.7]	Ref		
	≥2010	6	78.3 [67.8–88.9]	0.26		
Region	Western Africa	8	85.0 [78.1–91.9]	0.64		
	Eastern Africa	8	89.4 [84.9–93.9]	Ref		
	Southern Africa	11	81.0 [75.3–86.6]	0.11		
	Central Africa	4	83.2 [77.2–89.1]	0.51		
	Several region	1	62.3 [60.3–74.3]	0.010		
Type of site	Public sector	28	85.4 [81.6–89.3]	Ref	Ref	Ref
	Other	3	68.2 [60.9–75.5]	0.005	0.030	0.008
Number of sites	1	18	83.9 [78.0–89.8]	Ref		
	≥2	8	82.3 [77.2–87.3]	0.70		
Decentralized area	No	24	82.5 [78.1–86.9]	Ref		
	Yes	7	88.5 [83.7–93.3]	0.21		
Population selected	General	24	84.1 [79.8–88.4]	Ref		
	General, with FU >6 months	5	77.8 [67.5–88.1]	0.14		
VL monitoring before evaluation	No	11	82.6 [73.2–92.0]	Ref		
	Yes	21	84.3 [80.2–88.3]	0.67		
Tracking of LTFU patients	No	24	82.2 [77.4–86.9]	Ref		
	Yes	8	88.2 [82.7–93.8]	0.13		
Median age at ART initiation (years)	≤36	13	87.7 [83.3–92.2]	Ref	Ref	
	>36	15	80.3 [73.7–87.0]	0.044	0.18	
Proportion of women	≤65	12	79.1 [72.1–86.1]	Ref	Ref	Ref
	>65	20	86.5 [83.4–89.7]	0.020	0.29	0.09
Proportion of patients in WHO stage 3–4	≤69	11	83.3 [76.8–89.9]	Ref		
	>69	11	86.7 [82.2–91.2]	0.45		
Median CD4 at ART initiation (cells/mm^3^)	≤135	18	82.6 [78.1–87.1]	Ref		
	>135	12	85.8 [77.5–94.0]	0.41		
Median VL at ART initiation (log copies/mL)	≤5.2	5	91.7 [87.8–95.7]	Ref	Ref	
	>5.2	8	83.0 [75.5–90.5]	0.09	0.24	
Proportion of EFV exposed patients	≤40	13	82.5 [74.7–90.4]	Ref		
	>40	10	82.6 [75.6–89.7]	0.98		
Proportion of NVP exposed patients	≤60	13	79.7 [73.0–86.5]	Ref		
	>60	10	86.4 [81.3–91.6]	0.12		

Using an OT approach, 7 evaluations of virological success were available at 40 copies/mL threshold, 14 evaluations of virological success were available at 1000 copies/mL threshold and 8 evaluations of virological success were available at 5000 copies/mL. The overall proportions (95% CI) of virological success at these thresholds were 83.1% (75.5–90.6), 84.1% (79.2–88.9) and 91.1% (87.3–94.9), respectively (S1 Fig).

**Fig 5:**
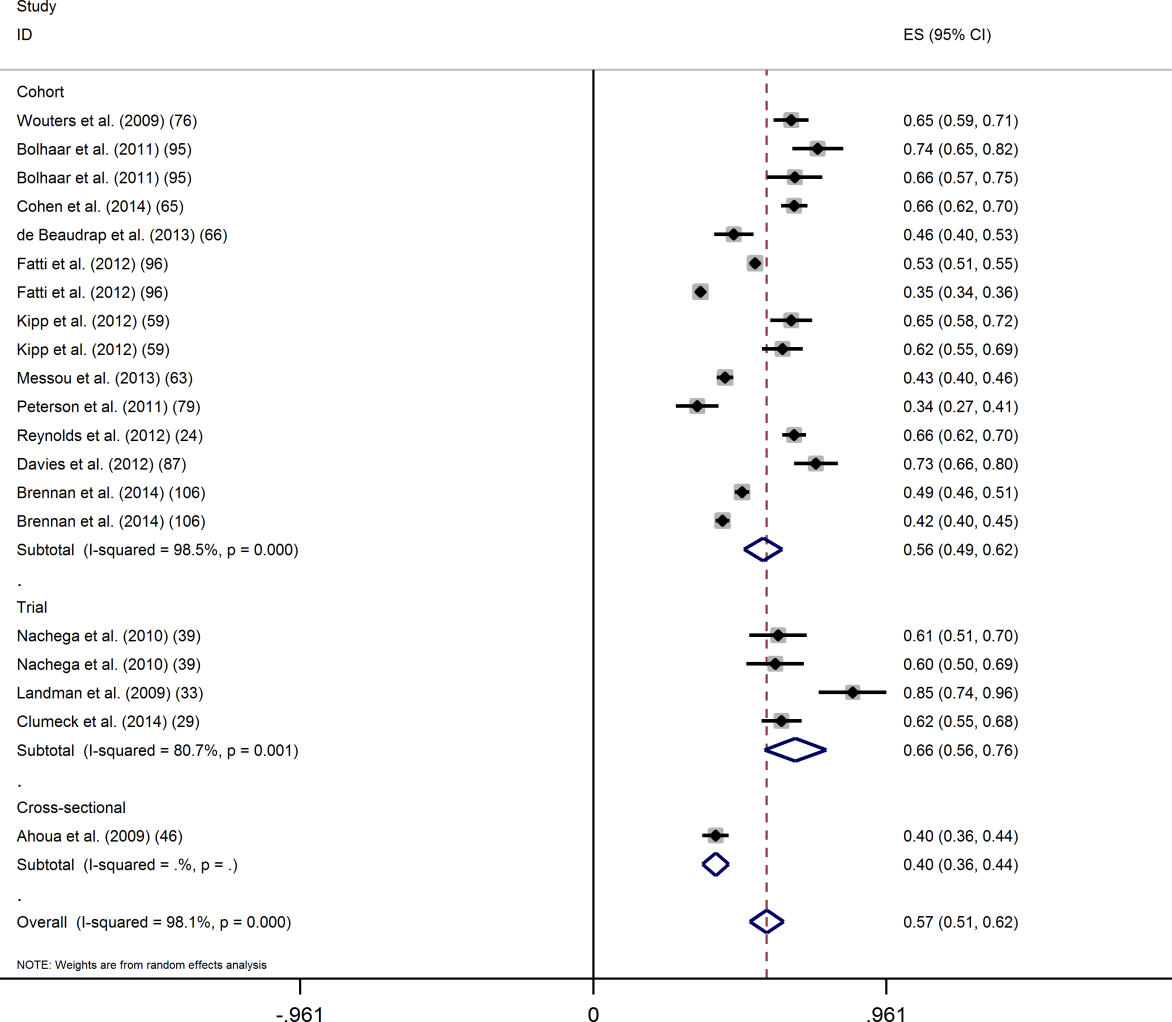
Intention-to-treat analysis of the proportion of virological success (400 copies/mL) at 24 months of ART by study design.

Using an ITT approach, 20 evaluations of virological success at 24 months after ART initiation at the threshold of 400 copies/mL were considered. The overall proportion of virological success (95% CI) was 56.8% (51.3–62.4) (Fig 5). Again, the Cochran test (p<0.001) and the I-square statistic (98.2%) indicated large between-study heterogeneity. In univariate analysis, the proportion of virological success tended to be higher in clinical trials than in cohort studies (Table 4). The proportion of virological success was significantly higher in studies set in the public sector, and in studies which did not track LTFU patients.

**Table 4:** Rate of virological success (<400 copies/mL) at 24 months–intention-to-treat analysis.

				Univariate	Multivariate	
				analysis	analysis	
Variable	categories	N	Rate (95% CI)	(p)	Full model (p)	Reduced model (p)
Study design	Cohort	16	54.6 [48.6–60.6]	Ref	Ref	
	Trial	4	66.2 [56.1–76.4]	0.14	0.88	
Beginning of enrolment	≤2005	14	58.5 [50.2–66.7]	Ref		
	≥2006	6	53.0 [46.9–59.2]	0.52		
End of enrolment	≤2005	4	58.7 [41.9–75.6]	0.92		
	2006–2009	12	60.7 [53.0–68.3]	Ref		
	≥2010	4	44.8 [35.4–54.3]	0.24		
Region	Western Africa	4	51.2 [37.6–64.8]	0.84		
	Eastern Africa	4	58.1 [44.3–72.0]	Ref		
	Southern Africa	11	58.0 [50.5–65.5]	0.99		
	Central Africa	1	61.7 [55.1–68.3]	0.99		
	Several region	-	-	-		
Type of site	Public sector	17	59.4 [53.7–65.2]	Ref	Ref	Ref
	Other	3	42.8 [29.4–56.2]	0.05	0.43	0.29
Number of sites	1	14	59.7 [52.9–66.5]	Ref		
	≥2	3	50.1 [39.3–60.8]	0.32		
Decentralized area	No	14	57.1 [50.9–63.4]	Ref		
	Yes	3	62.4 [57.6–67.2]	0.86		
	Both	2	44.1 [26.5–61.5]	0.39		
Population selected	General	15	55.3 [48.9–61.7]	Ref		
	General, with FU >6 months	3	51.2 [37.9–64.6]	0.62		
	Specific	-	-	-		
VL monitoring before evaluation	No	4	48.0 [35.8–60.3]	Ref		
	Yes	16	59.1 [53.2–65.0]	0.15		
Tracking of LTFU patients	No	13	61.6 [54.8–68.5]	Ref	Ref	Ref
	Yes	7	48.2 [39.6–56.8]	0.048	0.46	0.22
Median age at ART initiation	≤36 years	9	54.6 [45.4–63.8]	Ref		
	>36 years	9	56.1 [49.4–62.9]	0.78		

Using an ITT approach, 8 evaluations of virological success at 24 months after ART initiation were available at 40 copies/mL threshold and 6 evaluations of virological success were available at 1000 copies/mL threshold. The overall proportions of virological success (95% CI) were 57.6% (49.6–65.5) and 47.0% (36.1–58.0), respectively. Only one evaluation was performed at the threshold of 5000 copies/mL. This indicated a 67.3% proportion of virological success (95% CI: 63.4–71.2) [24] (S1 Fig).

### Virological success at M6

Using an OT approach, 13 evaluations of virological success at 6 months after ART initiation were available at the threshold of 40 copies/mL, 44 evaluations of virological success were available at the threshold of 400 copies/mL, 11 evaluations of virological success were available at the threshold of 1000 copies/mL, and 3 evaluations of virological success were available at the threshold of 5000 copies/mL. The overall proportions (95% CI) of virological success at these thresholds were 71.7% (62.8–80.7), 83.8% (81.0–86.6), 85.5% (81.7–89.3), and 92.7% (87.6–97.8), respectively.

Using an ITT approach, 7 evaluations of virological success at 6 months after ART initiation were available at the threshold of 40 copies/mL, 31 evaluations of virological success were available at the threshold of 400 copies/mL, 10 evaluations of virological success were available at the threshold of 1000 copies/mL and 2 evaluations of virological success were available at the threshold of 5000 copies/mL. The overall proportions (95% CI) of virological success at these thresholds were 65.0% (48.4–81.6), 68.5% (62.8–74.2), 70.9% (62.7–79.2) and 87.1% (83.6–90.5), respectively (S1 Fig).

### Virological success at M36

Using an OT approach, 12 evaluations of virological success at 36 months after ART initiation were available at the threshold of 400 copies/mL, 4 evaluations of virological success were available at the threshold of 1000 copies/mL, and 3 evaluations of virological success were available at the threshold of 5000 copies/mL. The overall proportions (95% CI) of virological success at these thresholds were 85.8% (82.2–89.3), 81.7% (77.9–85.5) and 92.8% (89.4–96.2), respectively.

Using an ITT approach, 5 evaluations of virological success at 36 months after ART initiation were available at the threshold of 400 copies/mL, and 2 evaluations provided proportions of virological success at the thresholds of 1000 copies/mL. The overall proportions (95% CI) of virological success at these thresholds were 37.8% (22.7–52.9) and 31.6% (0.03–59.8) (S1 Fig), respectively. Only one evaluation was performed at the threshold of 5000 copies/mL. This indicated a 63.3% proportion of virological success (95% CI: 59.3–67.3) [24] (S1 Fig).

#### Evaluation of publication bias

The funnel plots of the individual studies showed an asymmetry, with smaller studies reporting higher proportions of virological success more often (S2 Fig). Applying the method proposed by Eger and colleagues, the presence of publication bias was evidenced in the ITT analysis at 24 months (p-value for the intercept: 0.018), but not in any other analysis.

## Discussion

In this meta-analysis, we reported the proportion of patients in virological success while on first-line ART in sub-Saharan Africa, based on 85 recently published articles that led to 125 independent study populations comprised of 156,798 individuals. To our knowledge, this is the most recent and largest meta-analysis to compare virological success at specific time points in sub-Saharan Africa.

We did not observe an improvement in the proportion of virological success as compared to previous meta-analyses [8–10, 15, 17]. Our meta-analysis however is novel in the way in which we restricted our search to studies implemented in sub-Saharan Africa, and for the fact that it is not largely based on clinical trials. As compared to previously published meta-analyses which were not restricted by geographic location, and largely based on the results of clinical trials [15, 17], we believe that our meta-analysis is more representative of real-life treatment and care settings in sub-Saharan Africa–the region with the highest HIV burden.

In all the studies considered in this meta-analysis, the most common threshold for the evaluation of virological success was 400 copies/mL. Using this threshold and an OT approach, (e.g. only considering those still in care and on ART at the time of evaluation), the proportion of virological success was around 85%. Interestingly, this proportion of virological success remained stable regardless of the time point between M6 and M36. This result is likely due to the fact that only those who remain alive and in care are considered, which induces a healthy survivor bias–a bias which has been reported previously [17].

Several of the studies included in the meta-analysis also reported virological success at the threshold of 1000 copies/mL. This is the threshold for virological failure as defined by the latest WHO recommendations [110]. Using an OT approach, increasing the threshold from 400 to 1000 copies/mL did not increase the proportion of virological success by a significant amount, suggesting that few patients exhibit intermediate replicative levels. From a virological point of view, defining success at the threshold of 400 or 1000 copies/mL raises the potential for acquisition of drug resistance at a low level of replication [111, 112]. From a public health perspective, our results suggest that defining virological success at the threshold of 1000 or 5000 copies/mL is not identical, which is in accordance with the 2013 WHO recommendations in which the threshold defining virological success was lowered from 5000 to 1000 copies/mL [4]. Our results indicate that broadening access to viral load monitoring for patients under ART, especially through use of tools such as dried blood spots (which allows for a threshold of 1000 copies/mL, but not any lower) [113–116] should be more of a priority than lowering the threshold defining virological success. These findings support the WHO guidelines, in which virological failure remains at the threshold of 1000 copies/mL.

The results of our meta-analysis on the proportions of virological success at 6, 12, 24 and 36 months after ART initiation defined at the thresholds of 400 and 1000 copies/mL are comparable to the results from another meta-analysis performed in resource limited settings [17], despite this meta-analysis aggregating studies irrespective of the threshold.

Of the 125 study populations considered, only 28 (22.4%) directly reported the proportion of patients in virological success using an ITT approach. To better estimate the proportions of success, we used results when reported using an ITT approach, but also searched the articles for the number of deaths, loss to follow-up, switches to 2^nd^ line ART, transfers/withdrawals, and for the number of patients who did not have a virological evaluation. This enabled us to estimate the proportion of patients in virological success using an ITT approach in an additional 38 studies.

Regardless of the time point following ART initiation and the threshold used to define virological success, results using an ITT approach were lower than those using an OT approach. OT results evaluate ART efficacy for those still on ART, while ITT results evaluate the effectiveness of the programmes delivering ART, as those who died or were lost to follow-up are considered as failures, and these numbers increase with time. The difference in estimated proportions between the two approaches was substantial and as large as 15% at 6 months after ART initiation, reaching 20% at 12 months, and 25% at 24 months. This difference can be explained by death and patients LTFU, but also by the number of patients who did not have a virological evaluation performed. Studies in which the proportion of non-evaluated patients was >40% [96, 99, 100, 117, 118] were not considered in the ITT analysis, as we sought “systematic” virological evaluation, and it is possible that a lack of viral load monitoring was not random.

We wanted to investigate whether clinical trials, cohort studies, and cross-sectional studies provide similar quality information on proportions of virological success.

The fact that the proportions of virological success, using an OT approach, compare well across the various study designs indicates the effectiveness of ART in Africa. It also seems to indicate that patients in routine care are as adherent to ART as those enrolled in trials, even though the latter group may be more sensitised to this issue.

Cross-sectional studies, if based on a representative sample of the targeted population, have fewer logistical and financial constraints, allowing results to be obtained more quickly than cohort studies. Such studies are of special interest for national HIV treatment programmes, as they provide valuable data for the planning of future activities. They are also recommended by WHO for the surveillance of resistance in patients on ART [119].

On the other hand, using an ITT approach, the proportion of virological success was significantly higher in randomised trials than in cohorts at 12 months after ART initiation, and tended to be higher at 24 months. This could be explained by the selection of healthier participants, but also by more frequent monitoring of patients, more systematic clinical and biological examinations and by reinforced clinical teams in trials as compared to routine practice. All of these factors may have reduced attrition in clinical trials, and may explain the higher proportion of virological success as compared to cohort studies. Indeed, a relatively high mortality in the first 6 to 12 months after ART initiation and substantial loss to follow-up have been widely reported in resource limited settings [22, 23, 120, 121]. These results highlight the need to strengthen retention in care in these settings.

Unexpectedly, in ITT analysis there was no evidence of an effect of tracking of LTFU patients on the proportions of virological success at 12 months after ART initiation, and at 24 months the proportion of virological success was even lower in studies that tracked LTFU patients than in studies that did not. However, this result is likely influenced by two studies that implemented tracking, but which had very low proportions of virological success. In one of these studies, there was a very large (32%) proportion of deaths [79]. The other study was a multicentre nation-wide study in South Africa that reported particularly low ITT results [96]. This likely illustrates the heterogeneity in the definition of tracking of LTFU patients which ranges from limited telephone contact efforts to more intensive home visits. Despite this meta-analysis not finding a strong link between the tracking of LTFU patients and virological success, such initiatives should be more widely implemented as a means to maintain patients in care and to prevent the acquisition of resistance.

The proportion of patients in virological success in OT analysis was found to be larger in public health centres compared to other sites (faith-based, NGO supported or with a mixed recruitment). Public centres may have been selected based on their capacities to participate in such studies (e.g. sufficient number of medical staff, easy access to a biological laboratory), but these are not necessarily representative of all health centres in the country. Faith-based and NGO supported centres may treat lower socio-economic populations and data from these centres may be under-reported, leading to a publication bias. Motivations to publish may differ between centres, while the funnel plots used in this meta-analysis indicated that some small study bias may be present.

Some limitations of this meta-analysis should be noted. Studies considered were heterogeneous in their design but also in the populations they considered. Most studies were based on the general population with limited selection criteria, but some were restricted to specific populations, such as patients co-infected with hepatitis or tuberculosis, or women who had previously been exposed to ART for PMTCT, etc. Studies on these specific populations had a limited sample size, and were therefore thought to contribute to the heterogeneity among estimations. However, the effect of these specific populations was investigated and was not found to be significant.

The studies also differed in terms of their definition of virological success, partly due to the variability of the techniques used to measure viral load, which do not all have the same thresholds. To control for this variability, we performed estimations of virological success at various thresholds. In only a small number of studies was virological success based on the result of two viral load measurements. This may also have contributed to the heterogeneity of our results. However, the limited number of such studies meant that this effect on the meta-analysis could not be evaluated.

To explain the heterogeneity of virological success across various study populations, programme characteristics were investigated (e.g. tracking of LTFU patients, type of site, setting). However, no specific characteristics were identified. This may be explained by characteristics not being mentioned in the individual studies, resulting in the pooling of studies in the meta-analysis that were not in fact similar.

By definition, only studies with virological evaluation were considered in our meta-analysis. Many programs in Africa, where no viral load monitoring is available have thus not been considered in our meta-analysis. However, we do not anticipate that this is a source of bias in our results. Indeed, some of the studies we considered were virological evaluations at a specific time point, without routine viral load monitoring. This was particularly true for cross-sectional evaluations, nested in cohort studies, and we did not find evidence that results from cross-sectional evaluations differed from the other studies.

In conclusion, this is the largest, most up-to-date meta-analysis on proportions of virological success at specific time points after ART initiation and at multiple thresholds, based on both OT and ITT analysis and the first comparison between clinical trials, cohort studies, and cross-sectional studies. Evidence from this review suggests that the current international target to have 90% of patients on ART in virological success will require intensive strategies to improve adherence to ART, and retention in care. It is also important to improve access to viral load monitoring in order to detect those at risk of virological failure, as early as possible, offering them effective ART.

## Supporting information

S1 Checklist PRISMA 2009 checklist. (DOCX)

S1 Fig Estimation of the rate of virological success based on different threshold (in copies/mL) on on-treatment (full box) and intention-to treat (empty box) at: A) at 6 months of ART, B) 12 months of ART, c) 24 months of ART, and D) 36 months of ART.
(DOCX)

S2 Fig Funnel plots of the rate of virological success (defined at the threshold of 400 copies/mL) against sample size. A) OT analysis at 12 months; B) ITT analysis at 12 months; C) OT analysis at 24 months; D) ITT analysis at 24 months.
(DOCX)

S1 Table Quality evaluation of the articles based on the STROBE statement. (DOCX)
