# Supplementary material for: Virological success after 12 and 24 months of antiretroviral therapy in sub-Saharan Africa: Comparing results of trials, cohorts and cross-sectional studies using a systematic review and meta-analysis
Source: PLoS One. 2017 Apr 20;12(4):e0174767. doi: 10.1371/journal.pone.0174767 (PMC5398519; doi:10.1371/journal.pone.0174767)
Supplement: S1 Fig — Estimation of the rate of virological success based on different threshold (in copies/mL) on on-treatment (full box) and intention-to treat (empty box) at: A) at 6 months of ART, B) 12 months of ART, c) 24 months of ART, and D) 36 months of ART. (DOCX) [file pone.0174767.s002.docx]

Supplementary figure 1: Estimation of the rate of virological success based on different threshold (in copies/mL) on on-treatment (full box) and intention-to treat (empty box) at: A) at 6 months of ART, B) 12 months of ART, c) 24 months of ART, and D) 36 months of ART

1. At 6 months of ART
2. At 12 months of ART
3. At 24 months of ART
4. At 36 months of ART

ART: antiretroviral therapy; OT: on-treatment analysis; ITT: intention-to-treat analysis
