# Supplementary material for: Virological success after 12 and 24 months of antiretroviral therapy in sub-Saharan Africa: Comparing results of trials, cohorts and cross-sectional studies using a systematic review and meta-analysis
Source: PLoS One. 2017 Apr 20;12(4):e0174767. doi: 10.1371/journal.pone.0174767 (PMC5398519; doi:10.1371/journal.pone.0174767)
Supplement: S2 Fig — A) OT analysis at 12 months; B) ITT analysis at 12 months; C) OT analysis at 24 months; D) ITT analysis at 24 months. (DOCX) [file pone.0174767.s003.docx]

Supplementary figure 2: Funnel plots of the rate of virological success (defined at the threshold of 400 copies/mL) against sample size. A) OT analysis at 12 months; B) ITT analysis at 12 months; C) OT analysis at 24 months; D) ITT analysis at 24 months.

A)

B)

C)

D)
