# Supplementary material for: Virological success after 12 and 24 months of antiretroviral therapy in sub-Saharan Africa: Comparing results of trials, cohorts and cross-sectional studies using a systematic review and meta-analysis
Source: PLoS One. 2017 Apr 20;12(4):e0174767. doi: 10.1371/journal.pone.0174767 (PMC5398519; doi:10.1371/journal.pone.0174767)
Supplement: S1 Table — (DOCX) [file pone.0174767.s004.docx]

# *Supplementary data*

Supplementary Table 1: Quality evaluation of the articles based on the STROBE statement

| Ref | Specify study design | Describes the setting (country, number of sites, ART initiation dates) | Provides eligibility criteria | Provides details on virological evaluation (method, threshold) | Justify the sample size | Reports the number of participants at each stage of the study | Provides reasons for non participation at each stage of the study | Provides baseline characteristics of study participants | Reports the number of patients in virological success | Discusses limitations of the study |
| --- | --- | --- | --- | --- | --- | --- | --- | --- | --- | --- |
| Reynolds et al. (2012) ([24](#_ENREF_20)) | ✓ | ✓ | ✓ | ✓ |  | ✓ |  | ✓ | ✓ | ✓ |
| Goodall et al. (2014) ([25](#_ENREF_21)) | ✓ | ✓ (B) | ✓ | ✓ | ✓ (B) | ✓ | ✓ | ✓ | ✓ | ✓ |
| Munderi et al. (2010) ([26](#_ENREF_22)) | ✓ | ✓ | ✓ (B) | ✓ | ✓ | ✓ | ✓ | ✓ | ✓ | ✓ |
| Abdissa et al. (2014) ([27](#_ENREF_23)) | ✓ | ✓ | ✓ | ✓ | ✓ (B) | ✓ |  | ✓ (A) | ✓ | ✓ |
| Blacher et al. (2010) ([28](#_ENREF_24)) | ✓ | ✓ | ✓ (B) | ✓ (B) |  | ✓ |  | ✓ | ✓ | ✓ |
| Clumeck et al. (2014) ([29](#_ENREF_25)) | ✓ | ✓ | ✓ | ✓ | ✓ | ✓ | ✓ | ✓ | ✓ | ✓ |
| Jain et al. (2014) ([30](#_ENREF_26)) | ✓ | ✓ | ✓ |  |  | ✓ | ✓ | ✓ | ✓ | ✓ |
| Rositch et al. (2013) ([31](#_ENREF_27)) | ✓ | ✓ | ✓ | ✓ |  | ✓ |  | ✓ | ✓ | ✓ |
| Semvua et al. (2013) ([32](#_ENREF_28)) | ✓ | ✓ | ✓ | ✓ | ✓ |  |  | ✓ | ✓ | ✓ |
| Landman et al. (2009) ([33](#_ENREF_29)) | ✓ | ✓ | ✓ | ✓ |  | ✓ |  | ✓ |  |  |
| Bonnet et al. (2013) ([34](#_ENREF_30)) | ✓ | ✓ | ✓ | ✓ | ✓ | ✓ | ✓ | ✓ | ✓ | ✓ |
| Chang et al. (2010) ([35](#_ENREF_31)) | ✓ | ✓ | ✓ | ✓ | ✓ | ✓ |  | ✓ | ✓ | ✓ |
| Chung et al. (2011) ([36](#_ENREF_32)) | ✓ | ✓ | ✓ | ✓ |  | ✓ | ✓ | ✓ | ✓ (A) | ✓ |
| Laurent et al. 2011) ([37](#_ENREF_33)) | ✓ | ✓ | ✓ | ✓ | ✓ | ✓ | ✓ | ✓ | ✓ | ✓ |
| Lester et al. (2010) ([38](#_ENREF_34)) | ✓ | ✓ | ✓ | ✓ | ✓ | ✓ | ✓ | ✓ | ✓ | ✓ |
| Nachega et al. (2010) ([39](#_ENREF_35)) | ✓ | ✓ | ✓ | ✓ | ✓ | ✓ |  | ✓ | ✓ | ✓ |
| Taiwo et al. (2010) ([40](#_ENREF_36)) | ✓ | ✓ | ✓ | ✓ |  | ✓ | ✓ | ✓ | ✓ | ✓ |
| Van Loggerenberg et al. (2014) ([41](#_ENREF_37)) | ✓ | ✓ | ✓ | ✓ | ✓ | ✓ | ✓ | ✓ | ✓ | ✓ |
| Landman et al. (2014) ([42](#_ENREF_38)) | ✓ | ✓ | ✓ | ✓ | ✓ | ✓ | ✓ | ✓ | ✓ |  |
| Abdool Karim et al. (2011) ([43](#_ENREF_39)) | ✓ | ✓ (B) | ✓ | ✓ (B) | ✓ (B) | ✓ (A) |  | ✓ | ✓ (A) | ✓ |
| Mosam et al. (2012) ([44](#_ENREF_40)) | ✓ | ✓ | ✓ | ✓ | ✓ | ✓ (B) |  | ✓ |  | ✓ |
| Aghokeng et al. (2014) ([45](#_ENREF_41)) | ✓ | ✓ | ✓ | ✓ | ✓ | ✓ |  | ✓ | ✓ | ✓ |
| Ahoua et al. (2009) ([46](#_ENREF_42)) | ✓ | ✓ | ✓ | ✓ | ✓ | ✓ |  | ✓ | ✓ |  |
| Elul et al. (2013) ([47](#_ENREF_43)) | ✓ | ✓ | ✓ | ✓ | ✓ | ✓ |  | ✓ | ✓ | ✓ |
| Kouanfack et al. (2009) ([48](#_ENREF_44)) | ✓ | ✓ | ✓ | ✓ |  | ✓ |  | ✓ | ✓ | ✓ |
| McGuire et al. (2012) ([50](#_ENREF_46)) | ✓ | ✓ | ✓ | ✓ | ✓ | ✓ |  | ✓ (A) | ✓ (A) | ✓ |
| Hoffmann et al. (2009) ([49](#_ENREF_45)) | ✓ | ✓ | ✓ |  |  | ✓ |  | ✓ | ✓ | ✓ |
| Taieb et al. (2014) ([51](#_ENREF_47)) | ✓ | ✓ | ✓ |  |  | ✓ |  | ✓ | ✓ |  |
| Johannessen et al. (2009) ([52](#_ENREF_48)) | ✓ | ✓ | ✓ | ✓ |  | ✓ | ✓ |  | ✓ | ✓ |
| Aghokeng et al. (2013) ([53](#_ENREF_49)) | ✓ | ✓ | ✓ | ✓ |  | ✓ |  | ✓ | ✓(A) |  |
| Anude et al. (2013) ([54](#_ENREF_50)) | ✓ | ✓ | ✓ | ✓ |  | ✓ |  | ✓ | ✓ | ✓ |
| Chabikuli et al. (2010) ([55](#_ENREF_51)) |  | ✓ | ✓ |  |  | ✓ |  |  | ✓ |  |
| Dagnra et al. (2011) ([56](#_ENREF_52)) | ✓ | ✓ | ✓ | ✓ |  | ✓ |  | ✓ | ✓ |  |
| Dube et al. (2014) ([57](#_ENREF_53)) | ✓ | ✓ | ✓ |  | ✓ |  | ✓ | ✓ | ✓ | ✓ |
| Hong et al. (2013) ([58](#_ENREF_54)) | ✓ | ✓ | ✓ | ✓ |  | ✓ |  | ✓ | ✓ | ✓ |
| Kipp et al. (2012) ([59](#_ENREF_55)) | ✓ | ✓ | ✓ | ✓ | ✓ | ✓ | ✓ | ✓ | ✓ | ✓ |
| Kipp et al. (2010) ([60](#_ENREF_56)) | ✓ | ✓ | ✓ | ✓ | ✓ | ✓ | ✓ | ✓ | ✓ | ✓ |
| Alibhai et al. (2010) ([61](#_ENREF_57)) | ✓ | ✓ | ✓ | ✓ |  | ✓ |  | ✓ | ✓ | ✓ |
| Messou et al. (2011) ([62](#_ENREF_58)) | ✓ | ✓ | ✓ | ✓ |  | ✓ |  | ✓ | ✓ | ✓ |
| Messou et al. (2013) ([63](#_ENREF_59)) | ✓ | ✓ | ✓ | ✓ |  | ✓ |  | ✓ | ✓ | ✓ |
| Boulle et al. (2010) ([64](#_ENREF_60)) | ✓ | ✓ | ✓ | ✓ |  | ✓ | ✓ | ✓ | ✓ | ✓ |
| Cohen et al. (2013) ([65](#_ENREF_61)) | ✓ | ✓ | ✓ |  |  | ✓ | ✓ | ✓ | ✓ | ✓ |
| De Beaudrap et al. (2013) ([66](#_ENREF_62)) | ✓ | ✓ (B) | ✓ (B) | ✓ |  | ✓ (A) | ✓ (A) | ✓ | ✓ (A) | ✓ |
| De Luca et al. (2012) ([67](#_ENREF_63)) | ✓ | ✓ | ✓ | ✓ |  | ✓ | ✓ | ✓ |  |  |
| Ahonkhai et al. (2012) ([68](#_ENREF_64)) | ✓ | ✓ | ✓ |  |  | ✓ | ✓ | ✓ |  | ✓ |
| El-Hhatib et al. (2011) ([69](#_ENREF_65)) | ✓ | ✓ | ✓ | ✓ | ✓ (B) | ✓ |  | ✓ | ✓ | ✓ |
| Graham et al. (2010) ([70](#_ENREF_66)) | ✓ | ✓ | ✓ | ✓ |  | ✓ | ✓ | ✓ | ✓ | ✓ |
| Hamers et al. (2012) ([71](#_ENREF_67)) | ✓ | ✓ (B) | ✓ | ✓ |  | ✓ | ✓ | ✓ | ✓ | ✓ |
| Almeida et al. (2011) ([72](#_ENREF_68)) | ✓ | ✓ | ✓ | ✓ |  | ✓ | ✓ | ✓ | ✓ | ✓ |
| Jong et al. (2010) ([73](#_ENREF_69)) |  | ✓ | ✓ | ✓ |  | ✓ |  | ✓ |  | ✓ |
| Tiba et al. (2012) ([74](#_ENREF_70)) | ✓ | ✓ |  | ✓ |  | ✓ | ✓ | ✓ | ✓ | ✓ |
| Ugbena et al. (2012) ([75](#_ENREF_71)) | ✓ | ✓ | ✓ | ✓ |  | ✓ | ✓ | ✓ | ✓ | ✓ |
| Wouters et al. (2009) ([76](#_ENREF_72)) | ✓ |  | ✓ |  | ✓ | ✓ | ✓ (A) | ✓ | ✓ | ✓ |
| Koyalta et al. (2009) ([77](#_ENREF_73)) | ✓ | ✓ |  | ✓ |  | ✓ |  |  | ✓ | ✓ |
| Mutevedzi et al. (2010) ([78](#_ENREF_74)) | ✓ | ✓ | ✓ | ✓ |  | ✓ |  | ✓ | ✓ | ✓ |
| Peterson et al. (2011) ([79](#_ENREF_75)) | ✓ | ✓ | ✓ | ✓ |  | ✓ (A) |  | ✓ | ✓ (A) | ✓ |
| Polis et al. (2012) ([80](#_ENREF_76)) | ✓ | ✓ | ✓ | ✓ |  |  |  | ✓ | ✓ | ✓ |
| Rich et al. (2012) ([81](#_ENREF_77)) |  | ✓HIV care sites) | ✓ | ✓ |  | ✓ |  | ✓ | ✓ | ✓ |
| Rougemont et al. (2009) ([82](#_ENREF_78)) | ✓ | ✓ | ✓ | ✓ |  | ✓ | ✓ | ✓ | ✓ | ✓ |
| Rusine et al. (2013) ([83](#_ENREF_79)) | ✓ | ✓ | ✓ | ✓ |  | ✓ | ✓ | ✓ | ✓ | ✓ |
| Sivapalasingam et al. (2009) ([84](#_ENREF_80)) | ✓ | ✓ | ✓ | ✓ |  | ✓ |  | ✓ | ✓ | ✓ |
| Sunpath et al. (2012) ([85](#_ENREF_81)) | ✓ | ✓ | ✓ |  |  | ✓ | ✓ | ✓ | ✓ | ✓ |
| Billong et al. (2012) ([86](#_ENREF_82)) | ✓ | ✓ |  |  |  | ✓ | ✓ | ✓ | ✓ |  |
| Davies et al. (2012) ([87](#_ENREF_83)) | ✓ | ✓ | ✓ |  |  | ✓ |  | ✓ |  | ✓ |
| Innes et al. (2012) ([88](#_ENREF_84)) |  | ✓ |  |  |  | ✓ | ✓ | ✓ | ✓ |  |
| Billong et al. (2013) ([89](#_ENREF_85)) | ✓ | ✓ | ✓ | ✓ |  | ✓ | ✓ | ✓ | ✓ | ✓ |
| Aoudjane et al. (2014) ([90](#_ENREF_86)) | ✓ | ✓ |  | ✓ |  | ✓ | ✓ | ✓ | ✓ | ✓ |
| Nglazi et al. (2012) ([91](#_ENREF_87)) | ✓ | ✓ | ✓ |  |  | ✓ |  | ✓ | ✓ | ✓ |
| Nachega et al. (2009) ([92](#_ENREF_88)) | ✓ |  | ✓ |  |  | ✓ |  | ✓ | ✓ | ✓ |
| Wadonda-Kabondo et al. (2012) ([93](#_ENREF_89)) | ✓ | ✓ | ✓ | ✓ |  | ✓ | ✓ | ✓ | ✓ |  |
| Wadonda-Kabondo et al. (2012) ([94](#_ENREF_90)) | ✓ | ✓ | ✓ | ✓ | ✓ | ✓ | ✓ | ✓ | ✓ | ✓ |
| Bolhaar et al. (2011) ([95](#_ENREF_91)) | ✓ | ✓ | ✓ | ✓ |  | ✓ | ✓ | ✓ | ✓ | ✓ |
| Fatti et al. (2012) ([96](#_ENREF_92)) | ✓ | ✓ | ✓ |  |  | ✓ |  | ✓ | ✓ | ✓ |
| Huet et al. (2011) ([97](#_ENREF_93)) | ✓ | ✓ | ✓ (B) | ✓ |  | ✓ |  | ✓ |  | ✓ |
| Idoko et al. (2009) ([98](#_ENREF_94)) | ✓ | ✓ | ✓ | ✓ |  |  |  | ✓ |  | ✓ |
| Maskew et al. (2013) ([99](#_ENREF_95)) | ✓ | ✓ | ✓ |  |  | ✓ | ✓ | ✓ | ✓ | ✓ |
| Mbougua et al. (2010) ([100](#_ENREF_96)) | ✓ | ✓ | ✓ | ✓ |  | ✓ |  | ✓ |  | ✓ |
| Moore et al. (2010) ([101](#_ENREF_97)) | ✓ | ✓ | ✓ | ✓ | ✓ | ✓ | ✓ | ✓ | ✓ | ✓ |
| Mosha et al. (2013) ([102](#_ENREF_98)) | ✓ | ✓ | ✓ | ✓ | ✓ | ✓ | ✓ | ✓ | ✓ | ✓ |
| Franke et al. (2013) ([103](#_ENREF_99)) |  | ✓ | ✓ | ✓ |  | ✓ | ✓ | ✓ | ✓ | ✓ |
| Karasi et al. (2012) ([104](#_ENREF_100)) | ✓ | ✓ |  |  |  | ✓ |  | ✓ | ✓ |  |
| Shipton et al. (2012) ([105](#_ENREF_101)) | ✓ | ✓ | ✓ |  |  | ✓ |  | ✓ | ✓ | ✓ |
| Brennan et al. (2014) ([106](#_ENREF_102)) | ✓ | ✓ | ✓ |  |  | ✓ | ✓ | ✓ | ✓ | ✓ |
| Kassa et al. (2013) ([107](#_ENREF_103)) | ✓ | ✓ |  | ✓ |  | ✓ |  | ✓ |  |  |
| Igumbor et al. (2011) ([108](#_ENREF_104)) | ✓ | ✓ |  |  |  | ✓ |  | ✓ | ✓ | ✓ |

A- extra data obtained from the author

B- obtained completely or partially from another paper

C- Only percentages were provided
